# Hypertension Status and Associations with Self-Rated Health and General Practitioner Health Seeking in a Rural Australian Cohort

**DOI:** 10.3390/jcdd5040053

**Published:** 2018-11-06

**Authors:** Alex I. Gavino, Vivian Isaac, Craig S. McLachlan

**Affiliations:** 1 Rural Clinical School, Faculty of Medicine, University of New South Wales, Sydney, NSW 2052, Australia; algavino@gmail.com; 2 Flinders Rural Health South Australia, Flinders University, Renmark, SA 5341, Australia; vivian.isaac@flinders.edu.au

**Keywords:** blood pressure, hypertension, self-rated health, GP health-seeking

## Abstract

Hypertension is the most frequently managed condition by Australian general practitioners (GP). Knowledge of hypertension and blood pressure (BP) values may motivate individuals to seek GP management. Our study aims to determine the associations of knowledge of BP values, BP perception, GP health seeking, and self-rated health (SRH) in a rural population. Two-hundred and seventy-eight (278) residents responded to the health survey on socio-demographic profile, medical history, BP knowledge and perception, SRH, and GP visit frequency. Associations were evaluated using Chi-squared test and multivariate logistic regression. Cohort mean age was 63.6 (12.4) years with 63.3% females. Hypertension (37.8%) was the most common condition. GP visits were made at least once every month (19.1%), every 2–6 months (35.6%), >6 months (11.5%), or only when needed (29.5%). Univariate analyses showed age, education, alcohol consumption, comorbidities, hypertension status, and SRH were significantly associated with visit frequency. After adjustments, hypertension status (OR = 3.6, 95% CI [1.7, 7.9]) and poor SRH (OR = 3.1, 95% CI [1.4, 7.0]) were significantly associated with frequent monthly visits. Our cohort demonstrated that having hypertension and poor self-rated health were associated with frequent monthly GP visits. The perception of high blood pressure does not drive seeking additional GP input.

## 1. Introduction

Hypertension is the most frequently managed medical condition by general practitioners (GP) in Australia, responsible for 7.5% of patient encounters in practice [1]. Knowledge and awareness of blood pressure (BP) status likely influences a person’s BP control via GP review or therapeutic management [2,3]. However, a number of studies have demonstrated that community awareness of hypertension is limited [4,5,6]. In Australia, awareness of BP condition has been reported to be variable across rural populations. Janus and colleagues reported that 62.1% of hypertensive rural patients were aware of their high BP status [7], while another study reported a much lower level of awareness (43.4%) [8]. Whether BP knowledge and perception are associated with health seeking has not been reported in Australian rural populations.

Self-perception of health can be measured by an individual through a single-item question for self-rated health (SRH) [9,10]. SRH is a strong predictor of future health [10], health seeking [11], cardiovascular outcomes [12], disease morbidity [13,14], and mortality [14,15]. Moreover, poor SRH was found to be associated with more frequent utilization of hospital services [16]. Some current guidelines recommend the use of SRH status in combination with traditional risk factors (i.e., smoking status, hypertension, diabetes mellitus, and cholesterol) in determining cardiovascular risk of patients [12,15]. Although poor SRH is associated with hypertension incidence [13,17], hypertensive patients who reported excellent SRH exhibited better control of their BP [18]. Findings from two Australian population surveys have demonstrated that poor SRH is associated with increased use of general health services, including GP visits [19].

There is limited understanding of the relationship of knowledge of BP values and BP perception with GP health seeking, and whether the interaction is mediated by SRH. Our study aims to determine the associations of knowledge of BP values, perception of BP condition, GP health seeking, and SRH. Specifically, we aim to determine whether knowledge of BP values is associated with increased GP clinic visits.

## 2. Materials and Methods

### 2.1. Ethics Approval

The Human Research Ethics Committee of Ballarat Health Services and St. John of God Ballarat Hospital reviewed and approved the implementation of the Big Ararat Health Study. All participants provided written informed consent.

### 2.2. Participant Recruitment

Residents from Ararat Rural City in Western Victoria, Australia, aged 35 years old and over were invited to participate through public campaign involving newspaper articles and radio announcements. In 2016, it was estimated that 7376 residents were within that particular age group [20]. Advertisements and survey booklets were displayed prominently in public locations, including the library, schools, church, fitness centre, municipal offices, general practice clinics, hospital, community health centre, and local markets. Community engagement through information sessions with local socio-civic organisations and companies was also done. Residents who volunteered to participate were requested to fill-out the baseline survey booklet, including the attached consent form, and mail it to the study team through the return envelope provided. Return of the survey booklet with the signed consent form indicated their agreement to participate in the study.

### 2.3. Baseline Health Survey

The baseline health survey was a comprehensive questionnaire that covered participant demography, general health and wellbeing, nutrition, lifestyle, cardiovascular and neurocognitive health, self-rated health, and access to health services.

We recorded the participants’ sociodemographic characteristics, including age, sex, civil status, level and years of education, employment, smoking status, alcohol consumption, and living status. Current and past medical conditions were identified based on their self-report. We used the Charlson Comorbidity Index (CCI) as a measure of co-existing medical conditions that affected the participants. The CCI is a prognostic predictor of 10-year mortality and is based on the presence of co-morbidities [21]. Since hypertension was not part of the CCI calculation, we could use self-reported hypertension status separately in the analyses. We also asked participants to indicate their current medications from a list of commonly prescribed drugs. This would enable us to identify BP-lowering medications.

Specific questions pertinent for this study included BP knowledge and perception, SRH and GP health seeking. BP knowledge was defined as the participants’ knowledge of their actual BP values or status and was determined by asking: “Do you know your last measured blood pressure?” with responses: “I do not know”, “The doctor just said it was (low/normal/borderline/high)” or “Yes”. For those who knew their BP, the actual values were reported. BP perception was asked with the question: “How do you perceive your blood pressure: low, normal or high?”

SRH was determined using the standard question: “In general terms, how would you describe your overall health: Excellent, very good, good, fair, poor or not relevant?” The responses were further dichotomised to positive SRH (excellent/very good/good) or poor SRH (fair/poor). Those who did not provide any response or answered “not relevant” were excluded from the analysis.

GP health seeking was determined through the frequency of GP visits (“more than once a month”, “every month/6 months/1 year/3 years/5 years/10 years”, “only when I need help”, “prefer to see specialist directly”, or “never”). Responses were aggregated into the following categories: (a) at least once per month, (b) at least once in less than 6 months, (c) at least once in 6 months or longer, and (d) only when needed. Those who responded, “prefer to see specialist directly” or “never”, and those who did not provide any response were excluded from the analysis.

### 2.4. Statistical Analysis

Descriptive statistics using frequencies and percentages were used to describe the sociodemographic and baseline characteristics of the participants. Chi-squared test, or Fisher’s exact test when appropriate, were used to evaluate the associations of sociodemographic characteristics, clinical risk factors, BP awareness, BP perception and SRH across the categories of GP visit frequency. We used multivariate logistic regression models to explore confounders in the associations. All statistical analyses were performed using the Statistical Package for Social Science (SPSS) software version 24.0 (IBM Corp., Armonk, New York, NY, USA) and statistical significance was defined at a value of *p* < 0.05.

## 3. Results

We received filled survey booklets from 278 eligible residents of Ararat Rural City. Four other booklets were excluded because two respondents were below the minimum age requirement while the other two were duplicate submissions. For the duplicate submissions, the latest submissions were included in the analysis.

### 3.1. General Findings

Table 1 summarises the baseline sociodemographic and clinical characteristics of the respondents surveyed. The mean age (SD) of the cohort was 63.6 (12.4) years and 63.3% were women. Hypertension (37.8%) was the most common self-reported medical condition. Mean systolic and diastolic BP were 128.4 (21.9) mmHg and 76.0 (12.8) mmHg, respectively. Other related comorbid conditions included dyslipidaemia (30.6%), cancer (25.5%), depression (16.9%), anxiety (14.4%), diabetes (10.8%), and myocardial infarction (5.0%). The mean CCI was 2.7 (SD 2.0, range 0–9). Approximately 80% of participants had positive SRH (excellent, very good, good) while 18% had poor SRH (fair, poor). None of the participants responded “not relevant” to the SRH question.

### 3.2. BP Knowledge, Perception and Monitoring

We identified 105 (37.8%) participants who self-reported having had a previous diagnosis of hypertension. We also noted that among those who had known hypertension, 18 (17.1%) participants did not list any medications for high BP. Other characteristics are listed in Table 2.

BP measurements across participants were performed by GPs (73.0%) during clinic visits or through home BP monitors (25.9%). More than half (56.5%) of the participants had their last BP measured within the past month, with 22.7% occurring during the past week. There were 107 (38.5%) participants who knew their BP values and self-reported them, while the remaining participants were unaware of their BP values (14.0%) or were given an indication that their BP status was normal or abnormal (45.0%). Participants perceived their BP as normal (68.7%) or abnormal (high [14.0%] or low [5.8%]), independent of awareness of BP values. Among those who knew their BP values (*n* = 107), 70 (65.4%) participants had correctly identified their BP status based on their perception of their BP.

### 3.3. Frequency of GP Health Seeking

The most common reasons for GP visits were prescription refills (42.4%), health checks (22.3%), or feeling unwell (18.3%). One-third of the participants (35.6%) visited their GP at least once in two to six months, while another third (29.5%) consulted with their GP only when they needed help. More frequent visits of at least once per month were made by 19.1%, with 4.0% going to the GP clinic more than once a month. Only 11.5% had their GP visits less frequently of once in more than 6 months or longer. None of the participants indicated that they never visited their GP (see Table 1).

### 3.4. Factors Associated with GP Health Seeking Frequency

Table 3 shows the distribution of sociodemographic characteristics, clinical risk factors, BP knowledge and perception, and SRH across the categories of GP visit frequency. Univariate analysis showed that factors associated with frequent GP visits of at least once a month were age ≥ 65 years old (*p* = 0.02), less than 12 years of education (*p* = 0.02), non-consumption of alcohol (*p* = 0.001), comorbidity score ≥ 3 (*p* = 0.001), self-reported history of hypertension (*p* < 0.001), and poor SRH (*p* < 0.001). Conversely, factors related to ad hoc (only when needed) GP visits were age 35–64 years old (*p* = 0.03), comorbidity score 0–2 (*p* = 0.001), no history of hypertension (*p* < 0.001), and positive SRH (*p* = 0.02). Almost half (47.6%) of those with hypertension visited their GP at least once in 2–6 months compared to 30.5% without hypertension (*p* = 0.01). Similarly, 43.5% with comorbidity score ≥ 3 consulted with the GP in 2–6 months in contrast to 31.5% with 0–2 comorbidity score (*p* = 0.04). Less frequent visits of at least once in more than 6 months or longer was associated with male gender (*p* = 0.05), living alone (*p* = 0.04), comorbidity score of 0–2 (*p* = 0.02), no previous diagnosis of hypertension (*p* = 0.02), and positive SRH (*p* = 0.02). We did not find any significant associations between frequency of GP visits and smoking, BMI, knowledge of BP values, and BP perception.

Table 4 shows the multivariate logistic regression analyses which explored the effects of confounders on the associations between frequency of GP use, and self-reported hypertension. After adjusting for factors with significant associations in the univariate analyses, we found that only hypertension status (OR = 3.6, 95% CI [1.7, 7.9], *p* = 0.001) and poor SRH (OR = 3.1, 95% CI [1.4, 7.0], *p* < 0.01) were significantly associated with frequent monthly GP visits. That is, those who have hypertension and have poor perception of health were over three times more likely to visit their GP monthly compared to their counterparts. In addition, having hypertension was also associated with a two-fold increase in the likelihood of visiting the GP every 2–6 months (OR = 2.1, 95% CI [1.2, 3.8], *p* = 0.02). Finally, those with hypertension were less likely to have ad hoc GP visits (OR = 0.2, 95% CI [0.1, 0.5], *p* < 0.001).

## 4. Discussion

In a cohort of rural participants aged 35 years old and above, we found that the frequency of GP visits was influenced by overall health perception and hypertension status. Having hypertension was associated with an almost four-fold likelihood of frequent monthly visits and a two-fold likelihood of visiting the GP at least once in 2–6 months. Self-reported hypertension was associated with more frequent GP visits because of the mediation of poor SRH. Previous studies have demonstrated that poor SRH was associated with hypertension incidence [13,17]. As previously noted, hypertension was the most common condition managed in Australian general practice [1]. This may be due to the need for hypertensive patients to return to their GP for regular prescription refills and follow-up. Similar findings were observed in rural China and, in addition, prescription refills for hypertension medications was the second most common reason for encounters [22].

In our study we were unable to demonstrate an association between knowledge of BP values and GP visits. This differs from a previous study by Brindel et al. which showed that patients who frequently visited their GP (at least thrice a year) and had recent BP measurements were more likely to be aware of their hypertension [23]. In our sample population, even if BP was measured within the previous month for majority of the participants, less than 40.0% were knowledgeable of their BP values. This level of BP knowledge among rural residents was even lower than the UK survey that found that 42.0% of the general population knew their own BP values [2]. For our study, this may be explained by the patient’s reliance on their GP’s interpretation of their BP status, as was observed in 45% of the participants. While it is more convenient for patients to remember their BP status interpretation (i.e., low, normal or high), health professionals need to encourage their patients to know their actual BP values and what their ideal target BP should be [4,24]. It would be difficult for patients to achieve their target BP levels if they are unaware of their baseline values. Furthermore, this may be useful when considering the possibility of adjusting cut-off values for hypertension classification in the future. Recently, the American guidelines have lowered the threshold for hypertension diagnosis [25]. People who fall within the range of 130–139 mmHg systolic BP or 80–89 mmHg diastolic BP, previously considered normal [26], may be considered to already have stage 1 hypertension [25].

Our analyses found an association between poor SRH and frequency of GP visits. Our findings are consistent with a Taiwan study that found increased health service utilization among patients with poor SRH; however, they did not observe increased outpatient visits among those with hypertension [16]. Despite the similarity, we acknowledge that health systems vary greatly worldwide and there may be other factors influencing frequent utilization of health services. One-third of our participants regularly visited their GPs at least once every six months while another third only went to their GPs when they needed help. The less frequent GP visits may be due to the positive SRH reported by more than three quarters of the respondents. However, for those with high BP, regular GP visits and BP monitoring may be necessary to manage their hypertension better. One study found that the low GP attendance rate of hypertensive patients impairs effective control of BP [27]. If health visits are less frequent, GPs may need to maximise the time with their patients to cover health promotion aspects of BP management.

Hypertension was the most common chronic condition in our cohort, which was self-reported by 37.8% of respondents. Inclusion of those receiving BP medications increases the proportion to 39.2%. This rate is higher compared to estimates by other Australian general population studies (32.1–34.0%) [24,28,29]. Although the reasons are unclear, one possible explanation for this is that we used a higher age limit cut-off (i.e., 35 years and older) which has resulted in more respondents who are older and at higher risk for hypertension [28]. Furthermore, our participant recruitment was based on open invitation to participate, which may have led to certain groups to be more inclined to participate. When compared to a similar local study conducted in a rural community, we found that our findings were similar [30]. These studies may demonstrate a higher burden of hypertension in rural and regional areas. In Australia, awareness of hypertension status and adequate BP management and control seem to be poor and rural residence may further worsen these factors [7,31].

We noted a few (1.4%) participants who did not report previous hypertension despite being on BP-lowering medications. It is possible that these respondents were completely unaware of the medical conditions they were being treated for, or they just opted not to respond to the medical history checklist. Our findings are relatively lower compared to the study by Jelinek et al. which found that 6.4% of rural patients were unaware of being identified as hypertensive but were taking BP-lowering medications [30].

Our study has certain limitations. The study cohort was localised to a rural community from a specific geographical boundary and socioeconomic characteristic. Hence, our findings may not be generalizable to a wider population. Our recruitment strategy may have had some influence on the profile of our respondents. The baseline questionnaire required some time to respond to. Our analyses were only based on self-reported data and self-recall may have some limitations over time. This paper does not include actual BP measurements. Future studies could include objective findings from health assessments and correlate these with the survey responses. Most of our respondents were older or retired since they had more time to answer the survey. However, advanced age may predispose them to certain cardiovascular diseases, such as hypertension. On the other hand, we adjusted for age in multivariate models and hypertension remained a predominate reason for GP clinic seeking.

## 5. Conclusions

Our study demonstrated that self-reported hypertension was associated with more frequent GP visits because of the mediation of poor SRH. Although knowledge of BP values and BP perception were not related to GP visits, GPs may initiate their health education by informing patients of their BP values, and understanding their BP perception and SRH. These may help patients become more active in the management and control of their BP.

## Figures and Tables

**Table 1:** Baseline sociodemographic and clinical characteristics of study participants [*n* = 278].

	*n*	
Gender		
Male	102	36.7
Female	176	63.3
Age (years), mean ± SD	63.6 ± 12.4	
35–44	18	6.5
45–54	49	17.6
55–64	76	27.3
65–74	81	29.1
75–84	41	14.7
>85	13	4.7
Country of birth *		
Australia	247	88.8
Foreign-born	30	10.8
Civil status		
Single	24	8.6
Married/Partner	179	64.4
Divorced/Separated	39	14.0
Widowed	36	12.9
Living alone *		
No	212	76.3
Yes	59	21.2
Education * (years), mean ± SD	14.1 ± 4.4	
No school certificate	23	8.3
Primary school (Year 6)	4	1.4
Secondary school (Year 10)	54	19.4
Senior high school (Year 12)	27	9.7
Certificate or diploma	69	24.8
University degree or higher	92	33.1
Employment		
No/Retired	144	51.8
Yes	134	48.2
Smoking *		
Never	145	52.2
Previous smoker	105	37.8
Current smoker	16	5.8
Alcohol consumption *		
No	37	13.3
Yes	231	83.1
BMI * (kg/m^2^), mean ± SD	27.6 ± 5.9	
Normal	72	25.9
Overweight	98	35.3
Obese	53	19.1
Blood pressure * (mmHg), mean ± SD		
Systolic	128.4 ± 21.9	
Diastolic	76.0 ± 12.8	
Self-reported medical conditions *		
Hypertension	105	37.8
Dyslipidaemia	85	30.6
Cancer	71	25.5
Diabetes	30	10.8
Depression	47	16.9
Anxiety	40	14.4
Myocardial infarction	14	5.0
Charlson comorbidity index *, mean ± SD	2.7 ± 2.0	
0	37	13.3
1–2	110	39.6
3–4	77	27.7
≥5	54	19.4
Self-rated health *		
Poor	8	2.9
Fair	42	15.1
Good	84	30.2
Very good	114	41.0
Excellent	23	8.3
GP visit frequency *		
At least once per month	53	19.1
At least once in 2–6 months	99	35.6
At least once in more than 6 months	32	11.5
Only when needed	82	29.5
Prefer specialist	1	0.3

* Total percentage does not equal to 100% due to missing responses. Abbreviations: SD = standard deviation; BMI = body mass index; GP = general practitioner.

**Table 2:** Self-reported hypertension status and BP-lowering medications.

		*n* (%)
Hypertension (self-reported)		105 (37.8%)
BP-lowering medications reported	87 (20.7%)	
BP-lowering medications not reported	18 (17.1%)	
Hypertension (not self-reported) but with BP-lowering medications		4 (1.4%)
Total hypertension (self-reported) or on BP-lowering medications		109 (39.2%)

**Table 3:** Distribution of sociodemographic characteristics, clinical risk factors, BP knowledge, BP perception and SRH across the categories of GP visit frequency.

Variables	At Least Once a Month			At Least Once in 2–6 Months			At Least Once in More than 6 Months or Longer			Only When Needed		
	*n* = 53 (19.1%)			*n* = 99 (35.6%)			*n* = 32 (11.5%)			*n* = 82 (29.5%)		
	*n*	%	*p* Value	*n*	%	*p* Value	*n*	%	*p* Value	*n*	%	*p* Value
Gender												
Female	35	21.0	0.64	61	36.5	0.79	15	9.0	0.05	56	33.5	0.22
Male	18	18.2		38	38.4		17	17.2		26	26.3	
Age, years												
35–64	20	14.4	0.02	47	33.8	0.25	21	15.1	0.13	51	36.7	0.03
≥65	33	26.0		52	40.9		11	8.7		31	24.4	
Education, years												
0–12	25	26.0	0.02	37	38.5	1.00	8	8.3	0.16	26	27.1	0.32
≥ 13	20	13.5		56	37.8		22	14.9		50	33.8	
Living alone												
No	36	17.5	0.33	76	36.9	0.53	30	14.6	0.04	64	31.1	0.87
Yes	13	24.1		23	42.6		2	3.7		16	29.6	
Smoking												
No	26	18.6	0.38	53	37.9	0.15	19	13.6	0.70	42	30.0	0.74
Previous smoker	19	18.6		40	39.2		10	9.8		33	32.4	
Yes	5	33.3		2	13.3		2	13.3		6	40.0	
Alcohol												
No	15	41.7	0.001	10	27.8	0.27	4	11.1	1.00	7	19.4	0.12
Yes	36	16.1		86	38.6		27	12.1		74	33.2	
BMI												
Normal	14	19.7	0.36	27	38.0	0.67	9	12.7	1.00	21	29.6	0.98
Overweight	14	14.9		39	41.5		12	12.8		29	30.9	
Obese	13	24.5		18	34.0		6	11.3		16	30.2	
CCI												
0–2	17	12.1	0.001	44	31.4	0.04	23	16.4	0.02	56	40.0	0.001
≥ 3	36	28.6		55	43.7		9	7.1		26	20.6	
Hypertension												
No	17	10.4	<0.001	50	30.7	0.01	26	16.0	0.02	70	42.9	<0.001
Yes	36	35.0		49	47.6		6	5.8		12	11.7	
BP Knowledge												
No	30	19.2	0.64	56	35.9	0.60	16	10.3	0.44	54	34.6	0.10
Yes	23	21.7		42	39.6		15	14.2		26	24.5	
BP Perception												
Low BP	2	13.3	0.06	4	26.7	0.29	2	13.3	0.10	7	46.7	0.08
Normal BP	29	15.4		70	37.2		26	13.8		63	33.5	
High BP	13	31.0		20	47.6		1	2.4		8	19.0	
SRH												
Fair/Poor	21	44.7	<0.001	17	36.2	1.00	1	2.1	0.02	8	17.0	0.02
Good/Excellent	32	14.7		80	36.9		31	14.3		74	34.1	

Note: Column totals may not be equal to (*n*) due to missing responses. Abbreviations: BMI = body mass index; BP = blood pressure, CCI = Charlson Comorbidity Index; SRH = self-rated health.

**Table 4:** Multivariate logistic regression analyses for the association between GP visit frequency and sociodemographic characteristics, clinical risk factors, high BP perception and SRH *.

Variables	At Least Once a Month		At Least Once in 2–6 Months		At Least Once in More than 6 Months or Longer		Only When Needed	
	OR (95% CI)	*p* Value	OR (95% CI)	*p* Value	OR (95% CI)	*p* Value	OR (95% CI)	*p* Value
Age, years								
35–64	Ref		Ref		Ref		Ref	
≥65	0.8 (0.3–2.3)	0.69	0.9 (0.4–1.9)	0.81	1.0 (0.4–2.9)	0.98	1.2 (0.6–2.7)	0.59
Education *, years								
0–12	Ref		Ref		Ref		Ref	
≥13	0.6 (0.3–1.3)	0.22	1.1 (0.6–1.9)	0.86	1.9 (0.7–4.9)	0.21	1.0 (0.5–2.0)	0.91
Alcohol								
No	Ref		Ref		Ref		Ref	
Yes	0.5 (0.2–1.2)	0.10	2.2 (0.9–5.4)	0.07	0.5 (0.1–1.8)	0.30	1.2 (0.4–3.1)	0.78
CCI								
0–2	Ref		Ref		Ref		Ref	
≥3	1.6 (0.6–4.6)	0.34	1.7 (0.8–3.5)	0.16	0.5 (0.2–1.6)	0.27	0.6 (0.3–1.2)	0.15
Hypertension								
No	Ref		Ref		Ref		Ref	
Yes	3.6 (1.7–7.9)	0.001	2.1 (1.2–3.8)	0.02	0.4 (0.1–1.1)	0.08	0.2 (0.1–0.5)	<0.001
SRH								
Good-Excellent	Ref		Ref		Ref		Ref	
Fair-Poor	3.1 (1.4–7.0)	<0.01	0.9 (0.4–1.8)	0.76	0.2 (0.0–1.4)	0.11	0.6 (0.2–1.4)	0.24

* Adjusted for age, years of education, alcohol consumption, comorbidity, hypertension status, and SRH. Abbreviations: CCI = Charlson Comorbidity Index; OR = odds ratio; Ref = reference; SRH = self-rated health.

## References

[1] Britt H., Miller G., Henderson J., Bayram C., Harrison C., Valenti L., Pan Y., Charles J., Pollack A.J., Wong C. (2016). General Practice Activity in Australia 2015–2016.

[2] Slark J., Khan M.S., Bentley P., Sharma P. (2014). Knowledge of blood pressure in a U.K. general public population. J. Hum. Hypertens..

[3] Wizner B., Gryglewska B., Gasowski J., Kocemba J., Grodzicki T. (2003). Normal blood pressure values as perceived by normotensive and hypertensive subjects. J. Hum. Hypertens..

[4] Oliveria S.A., Chen R.S., McCarthy B.D., Davis C.C., Hill M.N. (2005). Hypertension Knowledge, Awareness, and Attitudes in a Hypertensive Population. J. Gen. Intern. Med..

[5] Centers for Disease Control and Prevention (2012). Vital signs: Awareness and treatment of uncontrolled hypertension among adults—United States, 2003–2010. MMWR Morb. Mortal. Wkly. Rep..

[6] Pereira M., Lunet N., Azevedo A., Barros H. (2009). Differences in prevalence, awareness, treatment and control of hypertension between developing and developed countries. J. Hypertens..

[7] Janus E.D., Bunker S.J., Kilkkinen A., Namara K.M., Philpot B., Tideman P., Tirimacco R., Laatikainen T.K., Heistaro S., Dunbar J.A. (2008). Prevalence, detection and drug treatment of hypertension in a rural Australian population: The Greater Green Triangle Risk Factor Study 2004–2006. Intern. Med. J..

[8] White F., Wang L., Jelinek H. (2009). Awareness and pharmacotherapy of hypertension in a rural community. Med. Princ. Pract..

[9] Martin L.G., Schoeni R.F., Freedman V.A., Andreski P. (2007). Feeling better? Trends in general health status. J. Gerontol. B Psychol. Sci. Soc. Sci..

[10] Schnittker J., Bacak V. (2014). The increasing predictive validity of self-rated health. PLoS ONE.

[11] Jatrana S., Richardson K., Crampton P. (2014). Is change in global self-rated health associated with change in affiliation with a primary care provider? Findings from a longitudinal study from New Zealand. Prev. Med..

[12] Rumsfeld J.S., Alexander K.P., Goff D.C., Graham M.M., Ho P.M., Masoudi F.A., Moser D.K., Roger V.L., Slaughter M.S., Smolderen K.G. (2013). Cardiovascular Health: The Importance of Measuring Patient-Reported Health Status. Circulation.

[13] Barger S.D., Muldoon M.F. (2006). Hypertension labelling was associated with poorer self-rated health in the Third US National Health and Nutrition Examination Survey. J. Hum. Hypertens..

[14] van der Linde R.M., Mavaddat N., Luben R., Brayne C., Simmons R.K., Khaw K.T., Kinmonth A.L. (2013). Self-Rated Health and Cardiovascular Disease Incidence: Results from a Longitudinal Population-Based Cohort in Norfolk, UK. PLoS ONE.

[15] Barger S.D., Cribbet M.R., Muldoon M.F. (2016). Participant-Reported Health Status Predicts Cardiovascular and All-Cause Mortality Independent of Established and Nontraditional Biomarkers: Evidence From a Representative US Sample. J. Am. Heart Assoc..

[16] Isaac V., McLachlan C.S., Baune B.T., Huang C.T., Wu C.Y. (2015). Poor Self-Rated Health Influences Hospital Service Use in Hospitalized Inpatients With Chronic Conditions in Taiwan. Medicine (Baltimore).

[17] Carlsson A.C., Nixon Andreasson A., Wandell P.E. (2011). Poor self-rated health is not associated with a high total allostatic load in type 2 diabetic patients--but high blood pressure is. Diabetes MeTab..

[18] Hong T.B., Oddone E.Z., Dudley T.K., Bosworth H.B. (2005). Subjective and objective evaluations of health among middle-aged and older veterans with hypertension. J. Aging Health.

[19] Mewton L., Andrews G. (2013). Poor self-rated health and its associations with somatisation in two Australian national surveys. BMJ Open.

[20] Australian Bureau of Statistics Ararat (RC) (LGA). http://stat.abs.gov.au/itt/r.jsp?RegionSummary&region=20260&dataset=ABS_REGIONAL_LGA&geoconcept=REGION&datasetASGS=ABS_REGIONAL_ASGS&datasetLGA=ABS_REGIONAL_LGA&regionLGA=REGION&regionASGS=REGION.

[21] Quan H., Li B., Couris C.M., Fushimi K., Graham P., Hider P., Januel J.-M., Sundararajan V. (2011). Updating and Validating the Charlson Comorbidity Index and Score for Risk Adjustment in Hospital Discharge Abstracts Using Data From 6 Countries. Am. J. Epidemiol..

[22] Liu Y., Chen C., Jin G., Zhao Y., Chen L., Du J., Lu X. (2017). Reasons for encounter and health problems managed by general practitioners in the rural areas of Beijing, China: A cross-sectional study. PLoS ONE.

[23] Brindel P., Hanon O., Dartigues J.-F., Ritchie K., Lacombe J.-M., Ducimetière P., Alpérovitch A., Tzourio C., Investigators F.T.C.S. (2006). Prevalence, awareness, treatment, and control of hypertension in the elderly: The Three City study. J. Hypertens..

[24] Cadilhac D.A., Kilkenny M.F., Johnson R., Wilkinson B., Amatya B., Lalor E. (2015). The Know Your Numbers (KYN) program 2008 to 2010: Impact on knowledge and health promotion behavior among participants. Int. J. Stroke.

[25] Whelton P.K., Carey R.M., Aronow W.S., Casey D.E., Collins K.J., Dennison Himmelfarb C., DePalma S.M., Gidding S., Jamerson K.A., Jones D.W. (2018). 2017 ACC/AHA/AAPA/ABC/ACPM/AGS/APhA/ASH/ASPC/NMA/PCNA Guideline for the Prevention, Detection, Evaluation, and Management of High Blood Pressure in Adults: Executive Summary: A Report of the American College of Cardiology/American Heart Association Task Force on Clinical Practice Guidelines. Hypertension.

[26] James P.A., Oparil S., Carter B.L., Cushman W.C., Dennison-Himmelfarb C., Handler J., Lackland D.T., LeFevre M.L., MacKenzie T.D., Ogedegbe O. (2014). 2014 evidence-based guideline for the management of high blood pressure in adults: Report from the panel members appointed to the eighth joint national committee (jnc 8). JAMA.

[27] Filippi A., Paolini I., Innocenti F., Mazzaglia G., Battaggia A., Brignoli O. (2009). Blood pressure control and drug therapy in patients with diagnosed hypertension: A survey in Italian general practice. J. Hum. Hypertens..

[28] Carrington M.J., Jennings G.L., Stewart S. (2010). Pattern of blood pressure in Australian adults: Results from a national blood pressure screening day of 13,825 adults. Int. J. Cardiol..

[29] Appleton S.L., Neo C., Hill C.L., Douglas K.A., Adams R.J. (2013). Untreated hypertension: Prevalence and patient factors and beliefs associated with under-treatment in a population sample. J. Hum. Hypertens..

[30] Jelinek H., Kolbe C., Wang L., Oxbrow D. (2008). Identification of hypertension and efficacy of treatment in a rural Australian population. Clin. Exp. Hypertens..

[31] Briganti E.M., Shaw J.E., Chadban S.J., Zimmet P.Z., Welborn T.A., McNeil J.J., Atkins R.C. (2003). Untreated hypertension among Australian adults: The 1999-2000 Australian Diabetes, Obesity and Lifestyle Study (AusDiab). Med. J. Aust..

